# Effects of iron-based materials on anaerobic digestion of thermally hydrolyzed sewage sludge: methane production and speciation of potentially toxic elements

**DOI:** 10.1007/s00449-025-03280-9

**Published:** 2026-01-10

**Authors:** Luiza Usevičiūtė, Tomas Januševičius, Vaidotas Danila, Mantas Pranskevičius, Aušra Mažeikienė, Alvydas Zagorskis, Eglė Marčiulaitienė

**Affiliations:** Research Institute of Environmental Protection, Vilnius Gediminas Technical University, Vilnius, 10223 Lithuania

**Keywords:** Anaerobic digestion, Methane, Thermally hydrolyzed sewage sludge, Zero-valent iron, Magnetite, Nanoparticles

## Abstract

This study investigated the effects of iron-based materials—microscale zero-valent iron (mZVI), nanoscale zero-valent iron (nZVI), and nanoscale magnetite (nano-Fe_3_O_4_, in two size ranges: 50$\:-$100 nm and 14$\:-$29 nm)—on the anaerobic digestion (AD) of thermally hydrolyzed sewage sludge (THSS). Batch experiments were conducted under mesophilic conditions with three dosages (5, 15, and 30 mg/g-TS) of each material. Methane production kinetics were analyzed using the modified Gompertz model. A sequential extraction procedure was used to assess the speciation of potentially toxic elements (PTEs), namely, Zn, Cu, Pb, Ni, and Cr, in the digestates. The results showed that both mZVI and nZVI enhanced cumulative CH_4_ production more than either size of nano-Fe_3_O_4_. The highest cumulative CH_4_ yields (223 mL/g-VS_added_), approximately 9% higher than the control, were achieved at nZVI dosages of 5 and 15 mg/g-TS. Among iron-based materials, nZVI most effectively shortened the lag phase (1.6-fold decrease at 15 mg/g-TS), whereas both sizes of nano-Fe_3_O_4_ had minimal effect (maximum 1.06-fold decrease for the 50–100 nm Fe_3_O_4_ at 30 mg/g-TS). The addition of mZVI and nZVI increased the mobility of Zn, Cu, and Ni in the digested THSS samples, while both nano-Fe_3_O_4_ materials reduced mobility of all studied PTEs. Overall, the results indicate a trade-off between enhanced methane production and environmental risk; mZVI and nZVI improve AD but increase PTE mobility, whereas nano-Fe_3_O_4_ mitigates PTE mobility with little or no effect on CH_4_ production.

## Introduction

The treatment of sewage sludge (SS), which originates from wastewater treatment plants (WWTPs), is currently an urgent issue in Europe. Approximately 10 million tons of SS (dry weight) are generated annually in the European Union [1], while in Lithuania alone, 50 thousand tons were produced in 2018 [2]. As SS production continues to increase, there is a growing need for sustainable and energy-efficient management strategies. Common SS management options include thermal treatment, aerobic or anaerobic digestion (AD), lime stabilization, and landfill disposal [3]. Treated SS is often applied in agriculture as a soil fertilizer due to its nutrient content and organic matter [4]. However, the excessive or uncontrolled use of SS in agriculture can lead to soil contamination with potentially toxic elements (PTEs), posing risks to the environment and human health. According to Mattenberger et al. [5], SS accumulates toxic substances originating from pollutants present in wastewater.

Among the available SS treatment methods, AD is gaining importance as an efficient and environmentally friendly technology that simultaneously treats organic waste and generates renewable energy [6]. During AD, sludge is stabilized, and unpleasant odors and pathogen levels are reduced [7]. In practice, however, biogas production is a complex and time-consuming process, during which only up to 50% of the volatile suspended solids in the SS are converted into biogas [8]. The main components of biogas are methane (CH_4_) and carbon dioxide (CO_2_). The calorific value of biogas is directly dependent on the CH_4_ concentration [9]. Biogas produced from AD of SS typically contains 50–70% CH_4_. Undesirable components can constitute as much as 50% of the total biogas volume: CO_2_ generally accounts for 30–50%, hydrogen sulfide (H_2_S) for 0.1–3%, and water vapor (H_2_O) for 2–7% [10]. The AD technology of SS needs to be improved to increase CH_4_ production and concentration [9]. Various physical and chemical methods have been applied to enhance the accessibility of substrates to anaerobic microorganisms [11]. Among these, thermal hydrolysis pretreatment (THP) is a technique that improves the AD efficiency of SS [12]. The THP works by applying heat and high pressure to break down the sludge structure and disrupt microbial cells, thereby enhancing the sludge’s biodegradability—especially of recalcitrant substances such as lignin and cellulose [13, 14]. It is acknowledged as an eco-friendly and efficient technology that improves the dewaterability of SS before transportation/disposal and controls pollutants such as pathogens and viruses [15]. Moreover, studies have shown that THP can change speciation of PTEs, thereby influencing their stability and environmental risks [16, 17]. For example, Zhang et al. [18] studied the effect of THP on the heavy metal speciation of sludge before and after THP. The authors found that thermal hydrolysis decreased the content of arsenic (As), copper (Cu), zinc (Zn), and nickel (Ni) in acid-soluble and reducible fractions [18]. The addition of specific supplements such as macro-nutrients (e.g., nitrogen, phosphorus (P)) and micro-nutrients (e.g., trace elements in the form of salts, oxides, or nanomaterials) to SS is another method to enhance AD [19]. Various iron-based materials have been applied in AD studies to enhance CH_4_ production [20]. Multiple studies have reported that iron-based materials, such as microscale zero-valent iron (mZVI), nanoscale zero-valent iron (nZVI), and nanoscale magnetite (nano-Fe_3_O_4_), positively affect CH_4_ yield during AD processes [8, 21]. The properties of iron-based materials, such as their size or valence state, can significantly affect the efficiency of AD process. Several studies have demonstrated that smaller sizes of iron-based additives result in higher cumulative CH_4_ yields compared with larger particles. For example, Chen et al. [21] compared the effects of different zero-valent iron (ZVI) particle sizes (milli-ZVI, mZVI, and nZVI) on the mesophilic AD of waste-activated sludge and found that the highest cumulative CH_4_ production (83.9 mL/g-VS) was achieved when using nZVI [21]. The study conducted by Hassanpourmoghadam et al. [8] investigated nano-Fe_3_O_4_ particles of two different sizes (12–18 nm and 50–100 nm). It was observed that the smaller particles had a greater impact on AD performance: they released more Fe^2+^ ions, which reduced the sulfide-induced inhibition of methanogenesis and increased the rate of sludge hydrolysis. Furthermore, the addition of 12–18 nm nano-Fe_3_O_4_ (120 mg/L) enhanced wastewater sludge digestion and methanogenesis, resulting in a 1.7-fold increase in CH_4_ yield [8]. Xu et al. [22] studied the effects of mZVI (200 μm) and nZVI (35 nm and 50 nm) on the AD of blackwater. Among the tested particles, the nZVI of both sizes showed greater improvement in CH_4_ production compared to mZVI particles [22]. Overall, the studies indicate that smaller particles appear to produce greater improvements in AD performance and CH_4_ yield compared to larger particles. This enhancement is mainly attributed to their larger specific surface area (SSA). However, the higher reactivity of smaller nanoparticles may also inhibit microbial activity [22]; therefore, determining an optimal dosage is essential. Among iron-based materials, ZVI and Fe_3_O_4_ are the most commonly studied; however, it remains unclear which form of iron is more effective in improving AD performance. For example, Liang et al. [23] investigated the influence of different iron forms (ZVI and Fe_3_O_4_) on the anaerobic co-digestion of SS and food waste and found that cumulative CH_4_ production in the ZVI-treated group was 1.24-fold higher than that in Fe_3_O_4_-treated group [23]. In contrast, Zhao et al. [24] reported that cumulative CH_4_ production was higher in the Fe_3_O_4_ group (1639 mL) than in the ZVI group (1530 mL) during the AD of waste-activated sludge (WAS) [24]. Therefore, further studies are required to clarify the influence of different iron forms on the AD of SS.

Improper use of digested SS in agriculture can cause significant secondary pollution, mainly due to the presence of PTEs such as lead (Pb), chromium (Cr), Ni, Cu, Zn, and other elements [25]. These elements are characterized by their potential toxicity, long-term persistence in the environment, and their tendency to accumulate in living organisms [26, 27]. As a result, they can contaminate soil and water resources, eventually entering the food chain and posing serious risks to human health. It is well known that conventional sludge treatment methods, such as AD, are not effective at removing PTEs from sludge. Therefore, the addition of iron-based materials prior to AD can be an effective strategy for PTE stabilization. Iron-based materials can reduce mobile forms of PTEs and thus mitigate the environmental risks associated with the use of SS. Moreover, a previous study has demonstrated that nZVI and nano-Fe_3_O_4_ can help to reduce the mobility of PTEs during the AD process [28].

To date, most studies have focused on the effects of iron-based materials on the AD of untreated SS, with little attention given to thermally pretreated substrates. Thermal hydrolysis is a well-established industrial-scale SS pretreatment technology that improves biogas production [14]. However, one of the disadvantages of THP is the formation of inhibitory compounds, such as melanoidins, which can suppress methanogenic activity and reduce CH_4_ yield during AD [13, 29]. The addition of iron-based materials to thermally hydrolyzed SS could stimulate methanogens, shorten the adaptation phase, and stabilize PTEs by altering their speciation. Nevertheless, there is a lack of research on the effects of iron-based materials on methane yield and on the distribution of PTE chemical forms in thermally hydrolyzed sewage sludge (THSS). Therefore, THSS was selected as the substrate for this study. The objectives of the work were (1) to analyze the effects of different iron-based materials—specifically mZVI, nZVI and nano-Fe_3_O_4_ of two size ranges (50–100 nm and 14–29 nm)$\:-$on biogas production during the AD of THSS collected from the Vilnius WWTP; and (2) to determine the changes in PTE (Pb, Cr, Ni, Cu, Zn) speciation, as well as the ability of iron-based materials to reduce their mobility.

## Materials and methods

### Substrate and inoculum

The THSS (used as a substrate) and anaerobically digested sludge (used as inoculum) were collected from a WWTP located in Vilnius, Lithuania. At the WWTP, SS is generated in primary and secondary sedimentation tanks. The settled sludge from these tanks is mixed and stored in a sludge holding tank. The sludge mixture is then thickened by centrifugation prior to thermal hydrolysis. After THP, the sludge mixture undergoes AD in sludge digesters. The physicochemical characteristics of the THSS, inoculum, and their mixture are presented in Table 1.

**Table 1:** Physicochemical characterization of the THSS, inoculum, and their mixture before anaerobic digestion

Characteristic	THSS	Inoculum	Mixture
pH	5.41 ± 0.10	7.69 ± 0.10	6.32 ± 0.10
EC (mS/cm)	3.01 ± 0.01	10.2 ± 0.0	4.66 ± 0.01
TS (%)	8.52 ± 0.02	4.25 ± 0.02	6.97 ± 0.02
VS (%)	5.11 ± 0.02	2.52 ± 0.02	4.17 ± 0.02
VS/TS (%)	60.0	59.3	59.8
COD (g/L)	83.0 ± 0.6	40.0 ± 0.2	68.0 ± 0.5
TC (%)	36.8 ± 0.1	31.1 ± 0.1	34.8 ± 0.1
TN (%)	5.25 ± 0.10	5.18 ± 0.10	5.22 ± 0.10
C/N	7.01	6.00	6.67

Thermally hydrolyzed sewage sludge (THSS), Electrical conductivity (EC), Total solids (TS), Volatile solids (VS), Total chemical oxygen demand (COD), Total carbon (TC), Total nitrogen (TN)

### Iron-based materials

Four types of commercially available iron-based materials were used in this study: mZVI, nZVI, and nano-Fe_3_O_4_ with two distinct size ranges (50–100 nm and 14–29 nm). Nano-Fe_3_O_4_ with a size of 50–100 nm and mZVI were purchased from Sigma-Aldrich (Darmstadt, Germany), while nano-Fe_3_O_4_ with a size of 14–29 nm was obtained from Nanografi Nano Technology (Ankara, Turkey). Air-stable nZVI powder (NANOFER STAR) was purchased from Nano Iron s.r.o. (Židlochovice, Czech Republic). This nZVI powder consists of surface-stabilized nZVI particles, which remain air-stable due to a thin layer of iron oxides coating their surface. According to information of suppliers, mZVI particle size ranged from 45 to 150 μm. The average particle size of nZVI was 59.8 ± 1.3 nm, with a SSA of 19.4 m^2^/g. The SSAs of nano-Fe_3_O_4_ (50–100 nm) and nano-Fe_3_O_4_ (14–29 nm) were 6–8 m^2^/g and > 80 m^2^/g, respectively. Before use, the nZVI powder was activated in an aqueous suspension by mixing one part of air-stable powder with four parts of deionized water.

### Batch experiment

**Fig. 1:**
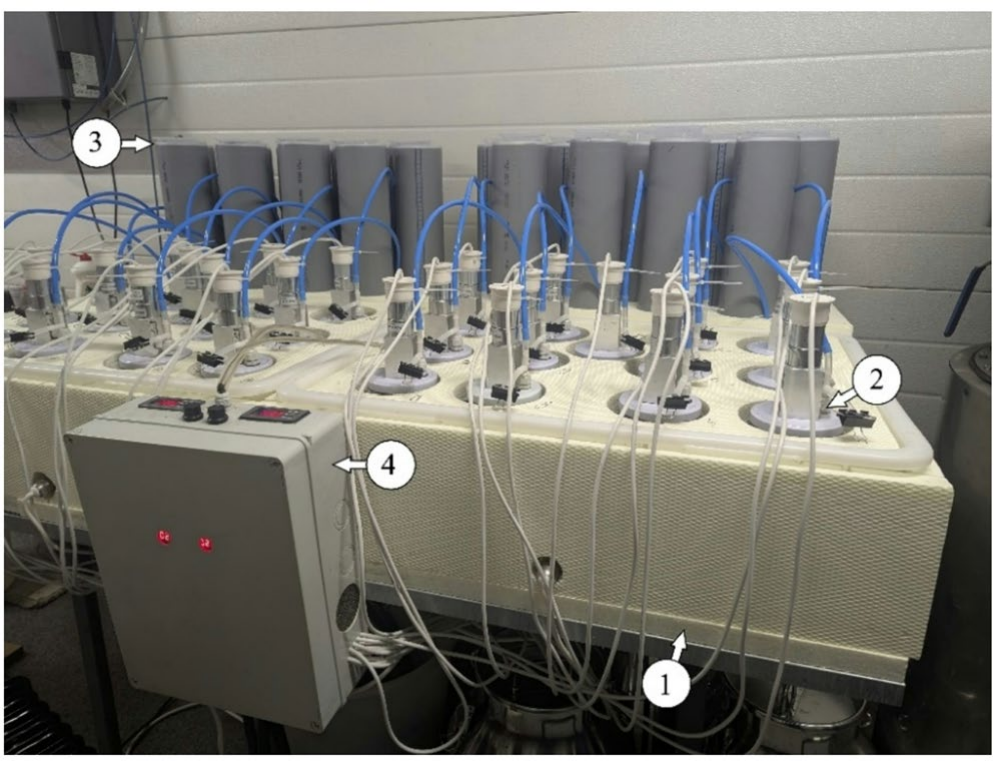
Laboratory-scale anaerobic digester system: (1) thermostatic water bath; (2) reactors with automated agitators; (3) biogas collection and measuring cylinder; and (4) device for temperature control

In order to evaluate the AD of THSS with iron-based additives, a laboratory-scale anaerobic digester system was designed (Fig. 1). The system consisted of 26 glass reactors (2.65 L each) equipped with automated agitators, a thermostatic water bath, and 2 L plastic measuring cylinders for biogas collection. The working volume of each reactor was 2 L, with an additional 0.65 L designated as headspace. Each reactor had two tubes: one used to assess gas composition, and the other connected to the biogas measuring cylinders to determine the volume of the produced gas. Before the AD, the THSS was mixed with inoculum at a ratio of 2:1 by volume (or 4:1 by VS), and the reactors were filled with the homogenized sludge mixture. To investigate the effects of iron-based materials on biogas and CH_4_ production, three dosages were tested: 5, 15, and 30 mg/g-TS. The mixtures were kept incubated for 40 days under mesophilic conditions (37 ± 1 °C). The performance of AD was evaluated by monitoring daily biogas production and analyzing its composition.

The operation parameters for each reactor are given in Table 2. Thirteen treatment groups were included in the experimental set-up and were labeled as B0 (control), B1 (with 5 mg/g-TS of mZVI), B2 (with 15 mg/g-TS of mZVI), B3 (with 30 mg/g-TS of mZVI), B4 (with 5 mg/g-TS of nZVI), B5 (with 15 mg/g-TS of nZVI), B6 (with 30 mg/g-TS of nZVI), B7 (with 5 mg/g TS of nano-Fe_3_O_4_ sized 50–100 nm), B8 (with 15 mg/g-TS of nano-Fe_3_O_4_ sized 50$\:-$100 nm), B9 (with 30 mg/g-TS of nano-Fe_3_O_4_ sized 50–100 nm), B10 (with 5 mg/g-TS of nano-Fe_3_O_4_ sized 14–29 nm), B11 (with 15 mg/g-TS of nano-Fe_3_O_4_ sized 14–29 nm), and B12 (with 30 mg/g-TS of nano-Fe_3_O_4_ sized 14–29 nm). To account for endogenous gas production, an inoculum-only blank (without THSS) was incubated in parallel. To obtain the actual biogas yield, the volume of biogas generated by the inoculum was subtracted from the total biogas volume measured in the other reactors. Two parallel experiments were performed for each treatment group.

**Table 2:** The operation parameters for each reactor

Reactor	Iron-based material	Dosage of iron-based material (mg/g-TS)	Particle size	Temperature (°C)	Initial pH
B0	–	0	–	37 ± 1	7.01 ± 0.10
B1	mZVI	5	45–150 μm		6.99 ± 0.10
B2		15			6.95 ± 0.01
B3		30			7.04 ± 0.04
B4	nZVI	5	59.8 ± 1.3 nm		7.07 ± 0.01
B5		15			7.17 ± 0.03
B6		30			7.21 ± 0.01
B7	nano-Fe_3_O_4_	5	50–100 nm		6.89 ± 0.01
B8		15			6.88 ± 0.00
B9		30			6.88 ± 0.01
B10		5	14–29 nm		6.88 ± 0.02
B11		15			6.88 ± 0.02
B12		30			6.87 ± 0.01

The pH of the sludge mixture was adjusted to 7.0 ± 0.2 using a NaOH solution (30%), providing optimal conditions for CH_4_-producing bacteria. The sludge was continuously mixed using stainless steel rods with a propeller, driven by agitator motors mounted on top of the digesters. The mixing speed was 120 rpm.

### Kinetic modeling

After completing the CH_4_ production test, the experimental data were modeled using a modified Gompertz model (MGM) (Eq. (1)) [30]. Modeling was performed to gain a better understanding of the impact of iron-based additives on CH_4_ production. This approach allowed for the estimation of three kinetic parameters: maximum CH_4_ production potential (*P*_*max*_), maximum CH_4_ production rate (*R*_*max*_), and lag phase (*λ*).

1 $$\:P\left(t\right)={P}_{max}\times\:exp\left[-exp\left\{\left.\frac{{R}_{max}\times\:e}{{P}_{max}}\times\:\left(\lambda\:-1\right)+1\right\}\right.\right],$$

where *P(t)*—cumulative CH_4_ production at time *t* (mL/g-VS_added_); *P*_*max*_—maximum CH_4_ potential (mL/g-VS_added_); *R*_*max*_—maximum CH_4_ production rate (mL/g-VS_added_/d); *λ*—lag phase (d); *t—*time (d); *e*—Euler’s number (2.72).

The kinetic parameters were obtained by optimizing a cost function aimed at minimizing the sum of squared errors between the model-predicted values and the experimental data [31]. This optimization was carried out using the Generalized Reduced Gradient non-linear solver in Microsoft Excel.

### Energy gain analysis

Energy gain was calculated using the calorific value (CV) of CH_4_ and the cumulative CH_4_ production obtained in the anaerobic batch experiments, as shown in Eq. (2) [32]:

2 $$\:{E}_{CH4}={V}_{CH4}\times\:{CV}_{CH4},$$

where *E*_*CH4*_—energy obtained from the combustion of CH_4_ produced (kJ); *V*_*CH4*_—cumulative CH_4_ volume produced during digestion (L); *CV*_*CH4*_—CV of CH_4_ expressed on a volumetric basis (kJ/L). The lower heating value of CH_4_ is 50 MJ/kg. To convert this mass-based value to a volumetric basis, it was multiplied by the density of CH_4_ at 37 °C and 1 atm (0.63 kg/m^3^), resulting in a CV of 31.5 kJ/L, which was applied in the calculations [33].

### Toxicity characteristic leaching procedure and sequential extraction procedure

To assess the mobility and speciation of PTEs, the Toxicity Characteristic Leaching Procedure (TCLP) and the modified Community Bureau of Reference (BCR) sequential extraction procedure were performed on the dry digested sludge samples, without and with iron-based materials. TCLP was applied to determine the leachable concentrations of Pb, Cr, Ni, Cu, Zn and P present in the digested sludge [34]. The TCLP method is primarily utilized to assess the leaching potential of elements under acidic conditions [35]. This method used an acetic acid-based extraction solution (pH = 4.93 ± 0.10). A total of 1 gram of the dried samples was weighed and placed in the 50 mL polypropylene copolymer centrifuge tubes. Twenty milliliters of TCLP solution were added to the centrifuge tubes, and the tubes were shaken for 18 h using a tube roller (RS-TR05, Phoenix Instrument, Germany). Then mixtures were centrifuged at 13,000 rpm using a centrifuge (Z 446, HermLe, Germany) for 15 min. Following centrifugation, the extracts were filtered through 0.45 μm cellulose acetate syringe filters and acidified with nitric acid. The TCLP leaching results were evaluated according to their corresponding non-hazardous limits for solid waste landfilling [35]. However, relying solely on the TCLP method to assess the long-term leaching behavior and stability of PTEs under adverse conditions is impractical [35]. Consequently, a modified BCR sequential extraction procedure was employed to determine the chemical speciation of PTEs in digested THSS samples. Four operationally defined fractions were determined: (F1) acid-soluble fraction—water and acid-soluble forms, including carbonates; (F2) reducible fraction—elements bound to iron/manganese oxides; (F3) oxidizable fraction—elements associated with organic matter and sulfides; and (F4) residual fraction—elements bound to non-silicate minerals (Table 3) [36]. Bioavailability of four fractions follows such order: F1 > F2 > F3 > F4 [37]. The first and second fractions are considered the most harmful to the environment, as they readily interact with plants and microbial communities and are generally associated with high bioavailability. In fact, it has been demonstrated that only elements in their free ionic or exchangeable forms can migrate through soil, accumulate in plant tissues, and directly harm the ecosystem [38]. The reducible fraction comprises the element content associated with iron and manganese oxides, which may become mobilized when the sludge is exposed to enhanced reducing conditions [39].

**Table 3:** Modified BCR sequential extraction procedure [40–42]

Step	Fraction	Mobility	Bioavailability	Reagents	Conditions
F1	Acid-soluble, bound to carbonate and cation exchange site	Mobile	Bioavailable	20 mL, acetic acid (0.11 M)	Shaking at room temperature, 16 h.
F2	Reducible, bound to Fe-Mn oxides	Mobile	Bioavailable	20 mL, hydroxylamine hydrochloride (0.5 M)	Shaking at room temperature, 16 h.
F3	Oxidizable, bound to organic matter and sulfides	Immobile	Potentially bioavailable	First, 10 mL of H_2_O_2_ (8.8 M), Next, 10 mL of H_2_O_2_ (8.8 M), Last, 50 mL of ammonium acetate	First, 1 h at room temperature and 1 h at 85 °C, Next, 1 h at 85 °C, Finally, shaking 16 h at room temperature.
F4	Residual, remaining residue, bound to mineral matrix	Immobile	Non-bioavailable	6 mL of aqua regia (HCl and HNO_3_)	Microwave digesting in a mixture of aqua regia at 180 °C.

For BCR extraction, 0.5 g of sludge sample was used. Between each step, the liquid phase was separated from the solid phase by centrifugation at 13,000 rpm for 20 min. The total contents of selected PTEs and P were determined by digesting 0.5 g of the dry THSS samples in aqua regia at 180 °C for 1 h using a microwave digestion system (Multiwave GO Plus, Anton Paar, Graz, Austria). The sums of the four PTE fractions showed good agreement with the total PTE concentrations, with recoveries ranging from 90.2 to 110%.

### Individual contamination factor and mobility factor

To evaluate the environmental risk of PTEs in digested THSS containing iron-based additives, two indicators were used: the individual contamination factor (ICF) and the mobility factor (MF). The ICF is a key indicator used to evaluate the potential environmental risk of a PTE based on its distribution among different geochemical fractions [43]. Meanwhile, the MF represents the percentage of PTEs in the most mobile and bioavailable fraction, helping to assess both environmental risk and the performance of element stabilization with iron-based materials [44]. The ICF was calculated by dividing the sum of the bioavailable and potentially bioavailable fractions (F1 + F2 + F3) by the non-bioavailable fraction (F4) for each sample. The MF was determined by dividing the acid-soluble fraction (F1) by the total of all four fractions (F1 + F2 + F3 + F4) [45]. All MF values were expressed as percentages.

### Analytical methods

Total solids (TS), volatile solids (VS), and total chemical oxygen demand (COD) were determined according to Standard Methods for the Examination of Water and Wastewater [46]. Total carbon (TC) and total nitrogen (TN) were determined using CHNS-O elemental analyzer (EA3000, EuroVector, Pavia, Italy). The pH and electrical conductivity (EC) were measured using a multi-parameter laboratory meter (Orion Versa Star Pro, Thermo Scientific, Waltham, MA, USA). Daily biogas volume was measured using water displacement method. Concentrations of CH_4_, CO_2_, H_2_, and H_2_S were measured using a portable gas analyzer (GAS DATA, GFM 406, Coventry, UK) to monitor changes in biogas composition. Specific CH_4_ and biogas yields in the system were determined based on the measured gas volumes and the added VS contents. The dissolved concentrations of PTEs and P were determined using an inductively coupled plasma optical emission spectrometer (ICP-OES, Avio 220 Max, PerkinElmer, USA). A TruQ multi-element standard solution containing Cr, Cu, Ni, Pb, and Zn and a TruQ single-element standard solution containing P were used for the preparation of calibration solutions (PerkinElmer). Analytical blanks were used with each set of samples to confirm the precision of the measurements.

### Statistical analysis

Physicochemical analyses were conducted in triplicate, while biogas experiments were performed in duplicate. Values were reported as the mean ± standard deviation (SD). To evaluate the experimental data, a one-way analysis of variance (ANOVA) was conducted to identify any statistically significant differences in TS, VS, COD, biogas, and CH_4_ production for different treatments [30]. A *p* value of 0.05 or lower indicates sufficient evidence to reject the null hypothesis. The coefficient of determination (*R²*), RMSE, and NRMSE were employed to evaluate the accuracy of the predictions, which were made using the MGM. All statistical analyses, model fitting and figure generation were performed using Microsoft Office Excel 2021.

## Results and discussion

### Effects of iron-based materials on biogas, CH_4_, CO_2_, H_2_S, and H_2_ production

**Fig. 2:**
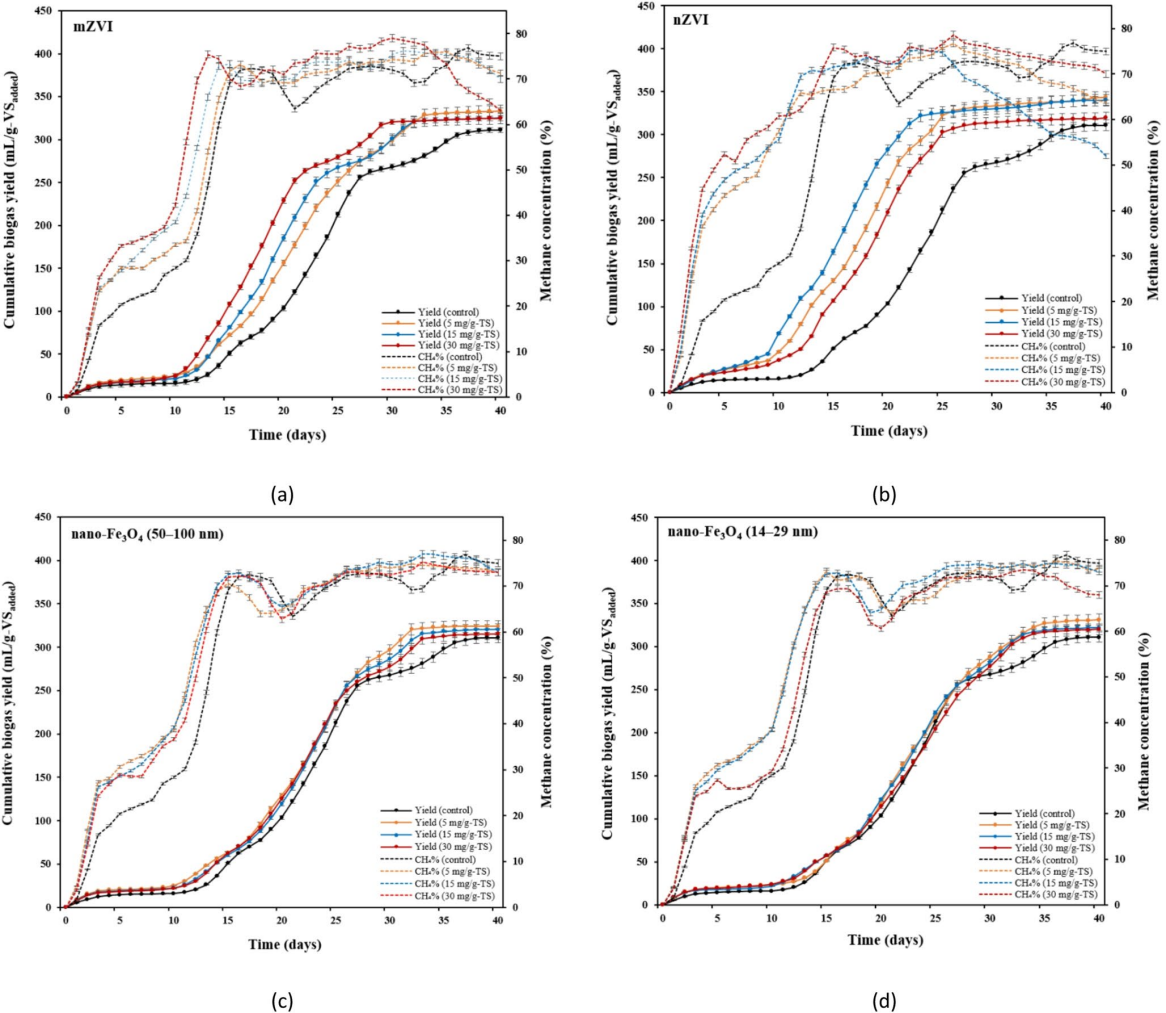
Effects of iron-based materials on cumulative biogas yield and methane concentration at various dosages: **a** mZVI; **b** nZVI; **c** nano-Fe_3_O_4_ (50–100 nm); and **d** nano-Fe_3_O_4_ (14–29 nm)

The maximum cumulative biogas yields (CBYs) were achieved in the THSS mixtures supplemented with 5 mg/g-TS and 15 mg/g-TS nZVI, reaching 343 mL/g-VS_added_ and 340 mL/g-VS_added_, respectively (Fig. 2b). These values were 10.3% and 9.33% higher than that observed in the control (*p* ≤ 0.05). An increase in biogas yield with nZVI addition was also observed by Lizama et al. [47]. These authors determined an increase in biogas yield from 132 mL/g-VS (control) to 310 mL/g-VS with the addition of 9 mg/g-VS nZVI. Jia et al. [48] also reported that the addition of nZVI to sludge promoted biogas production; 500 mg/L and 1000 mg/L nZVI dosages enhanced biogas production by 7.3% and 18.1%. It was concluded that nZVI dosage of 1000 mg/L could promote biogas production due to better living environmental parameters for methanogenic bacteria, such as pH value, concentrations of volatile fatty acids (VFAs), and ammonia nitrogen. The improvement in biogas production from the addition of nZVI particles to SS can be attributed to their strong catalytic properties, which enhance redox reactions essential to the AD process and influence the composition of microbial communities [49]. By promoting the decomposition of complex organic matter, they help increase the overall efficiency of biogas production. This catalytic effect is especially noticeable during periods of heightened biogas production, typically occurring after microorganisms have adapted to the presence of nZVI.

Comparing the two size ranges of the nano-Fe_3_O_4_, the lowest dosage (5 mg/g-TS) of smaller-size nanoparticles (14–29 nm) had a greater positive impact on CBY, which was 6.43% higher than that of the control (Fig. 2d). Meanwhile, larger nano-Fe_3_O_4_ (50–100 nm) had a lesser effect on biogas production, with the yield being only 4.18% higher compared to the control at the same dosage (Fig. 2c). The size of nano-Fe_3_O_4_ has been identified in previous studies as a critical factor in enhancing AD.

**Fig. 3:**
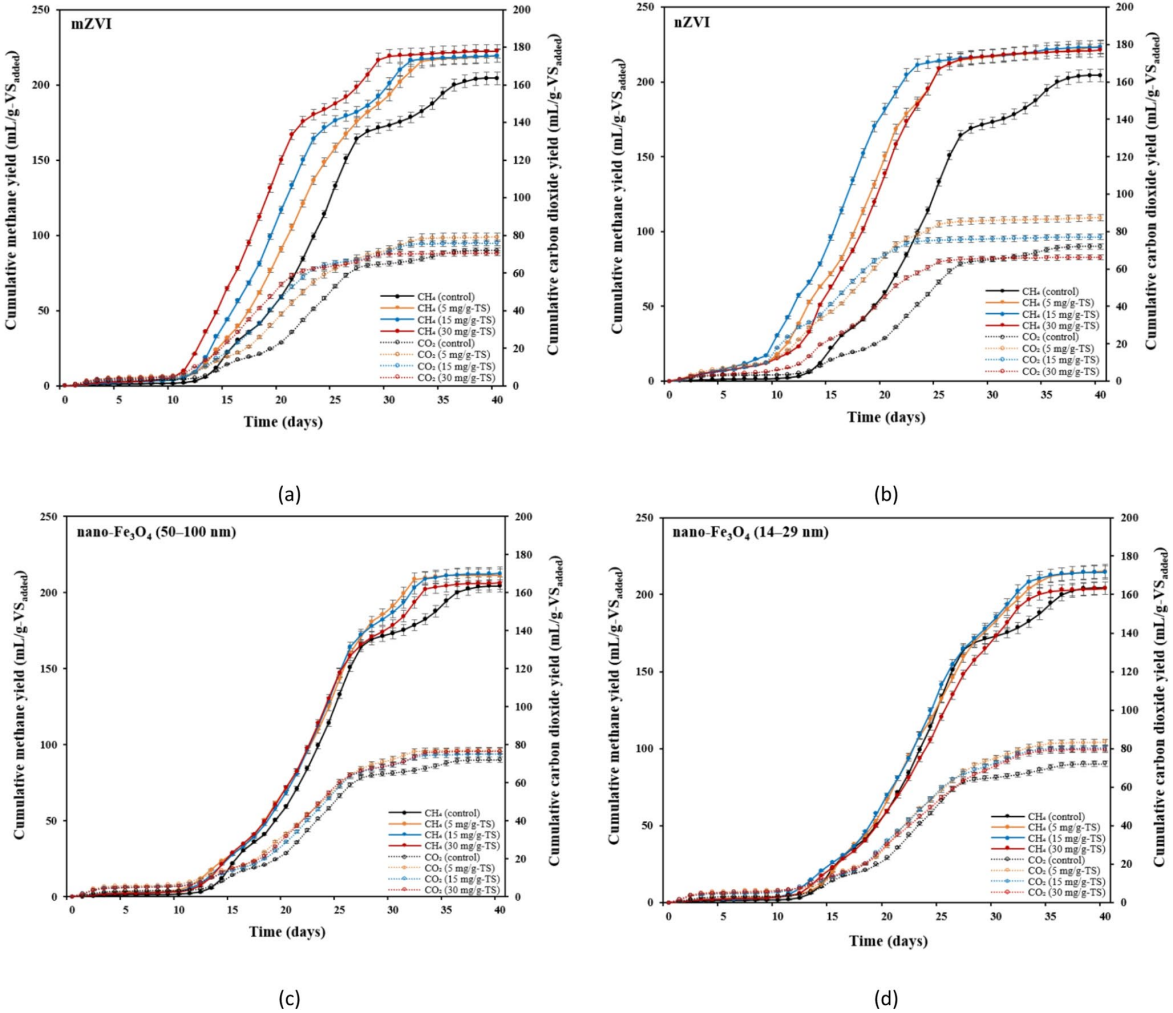
Effects of iron-based materials on cumulative methane and carbon dioxide yields at various dosages: **a** mZVI; **b** nZVI; **c** nano-Fe_3_O_4_ (50–100 nm); and **d** nano-Fe_3_O_4_ (14–29 nm)

Figure 3 presents CH_4_ production at varying iron-based material dosages. It was observed that both mZVI and nZVI particles had a greater effect on increasing the cumulative methane yield (CMY) compared to both sizes of nano-Fe_3_O_4_. The highest CMYs, obtained at nZVI dosages of 5 and 15 mg/g-TS, were 223 mL/g-VS_added_, representing a 9.31% increase compared to the control (Fig. 3b). The variation in cumulative CH_4_ yield across different nZVI dosages was minimal, suggesting that higher nZVI dosages had no substantial effect on enhancing CH_4_ production during the later phase of AD. In the case of mZVI, methane yields gradually increased from 204 to 219, 219, and 222 mL/g-VS_added_ with increases in dosages from 0 to 5, 15, and 30 mg/g-TS, respectively (Fig. 3a). The observed increase in cumulative CH_4_ yield with the addition of mZVI and nZVI was likely due to H_2_ generation through the H_2_ evolutional corrosion of these materials, which, in turn, stimulated CH_4_ production. Another study performed by Zhou et al. [50] reported that among the tested nZVI concentrations (10, 20, and 30 mM), the group treated with 30 mM nZVI achieved the highest cumulative CH_4_ production, showing a 37.5% increase compared to the control. Zhang et al. [51] found similar results, as micro-scale iron powder (200 μm), at a dosage of 10 g/L, increased CH_4_ production from 146 to 165 mL/g-VSS compared with the control during AD of WAS. Zhao et al. [24] reported that adding ZVI (200 μm) at a dosage of 10 g/L increased CH_4_ production by 21.3% when the same substrate was digested. ZVI was proposed to enhance WAS AD by promoting hydrogenotrophic methanogenesis since CH_4_ production via this pathway in the ZVI-amended reactor was 70% higher than in the control. According to another study by Wang et al. [52], the addition of 4 and 10 g/L micro-scale ZVI (100 mesh) enhanced CH_4_ production by 10.4% and 20.6% during AD of WAS, respectively, although these effects were less pronounced than those achieved with 1.0 g/L nZVI. A much higher dosage of mZVI (approximately nine times greater) was required compared with nZVI to achieve a similar enhancement of CH_4_ production, likely due to its lower reactivity and efficiency in improving the biochemical methane potential of WAS. Compared with mZVI, the markedly higher surface area of nZVI contributes to its greater ability to enhance AD as nZVI can more readily diffuse into cells and directly influence protein (enzyme) production [22, 53].

The results showed that the largest dosages (30 mg/g-TS) of both sizes of nano-Fe_3_O_4_ had no significant effect on CMY (Fig. 3c, d). On the other hand, when comparing nano-Fe_3_O_4_ of two different sizes, the smaller 14–29 nm particles resulted in slightly higher cumulative CH_4_ production than the larger ones (50–100 nm) at lower dosages (Fig. 3c, d). The nano-Fe_3_O_4_ (14–29 nm) at 5 and 15 mg/g-TS resulted in a 1.05-fold increase in CMY, while nano-Fe_3_O_4_ (50–100 nm) led to a 1.03–1.04-fold increase at the same dosages. This is in agreement with another study, which showed that 120 mg/L of the nano-Fe_3_O_4_ (12$\:-$18 nm) produced higher rise in CMY, i.e., about 1.7 times greater than the control, while 250 mg/L of 50$\:-$100 nm particles showed a 1.4-fold increase [8]. Such results, which showed that nano-Fe_3_O_4_ sized 12$\:-$18 nm had a more pronounced effect on CH_4_ production, were attributed to the greater SSA of the smaller nanoparticles. Additionally, smaller particles of nano-Fe_3_O_4_ are more likely to release soluble Fe²⁺, which can help reduce sulfide inhibition during methanogenesis, enhance the hydrolysis of solid substrates, or promote electron transfer between microbial species.

As the dosage of iron-based materials was increased from 0 to 5 mg/g-TS, the cumulative CO_2_ production showed a notable rise, reaching 79.0, 87.3, 76.8, and 83.3 mL/g-VS_added_ for mZVI, nZVI, nano-Fe_3_O_4_ (50–100 nm), and nano-Fe_3_O_4_ (14–29 nm), respectively, compared to the control (72.1 mL/g-VS_added_) (Fig. (3)). A similar tendency was found in the study by Feng et al. [54], where increasing the ZVI (200 μm) dosage from 0 to 4 g/L led to a rise in CO_2_ production from 124 to 146 mL/g-VSS. This increase occurred because CO_2_ is a byproduct of the sludge hydrolysis–acidification process [54]. However, when the dosage of mZVI and nZVI was further increased from 5 to 30 mg/g-TS, cumulative CO_2_ production decreased. This reduction in CO_2_ yield is attributed to the lower CO_2_ concentration observed at higher dosages of mZVI and nZVI.

The enhancement of CH_4_ yield by iron-based materials is associated with increased CH_4_ concentration in biogas production [55]. During days 3 to 6, CH_4_ content ranged from approximately 15.8% to 52.4% among the different groups, with the highest CH_4_ percentage observed in the group receiving a nZVI dosage of 30 mg/g-TS. By day 15, CH_4_ contents had stabilized at around 72% in nearly all reactors, except for B4 and B12, and stayed relatively consistent through the remaining AD process, ending on day 40. Over the entire digestion process, CH_4_ content in the biogas increased in all reactors following the addition of iron-based materials compared to the control reactor (B0) (Table 4). The maximum CH_4_ concentrations (around 79%) were obtained in two reactors—B3 and B6—where the highest dosages (30 mg/g-TS) of mZVI and nZVI were added. The addition of 30 mg/g-TS of nano-Fe_3_O_4_ (14–29 nm) resulted in the lowest average CH_4_ content (55.2%), which was similar to the control (54.9%). In contrast, the highest nZVI dosage of 30 mg/g-TS in this study resulted in the greatest increase in the average CH_4_ content of 21.1%, with the average value in reactor B6 reaching 66.5%. These results are similar to those of another study conducted by Niu et al. [56], in which the addition of 25 mg/g-TS of nZVI to SS in continuous reactors increased CH_4_ production by 27.6% compared to the control (from 34.8% to 44.4%). The study by Xu et al. [22] showed that both nZVI (35 nm) and mZVI (200 μm), at a dosage of 10 g/L, significantly increased CH_4_ content during AD of blackwater compared to the control (by 23.8% and 16.6%, respectively). The rise in CH_4_ concentration in the biogas with the addition of nZVI could be attributed to several factors. One possibility is the partial conversion of CO_2_ to CH_4_ through electron transfer processes. The microbial corrosion of nZVI may release electrons or generate dissolved H_2_, both of which can be utilized by hydrogenotrophic methanogens to reduce CO_2_ to CH_4_ in the final stage of AD [49, 57]. Additionally, homoacetogenic bacteria may use available H_2_ and CO_2_ to produce acetate, which can subsequently be converted to CH_4_ by acetotrophic methanogens [58].

**Table 4:** Methane and carbon dioxide contents from B0–B12 mesophilic reactors

Reactor	Maximum CH_4_ content (%)	Average CH_4_ content (%)	CH_4_ content (%) on day 15	Average CO_2_ content (%)
B0	76.8	54.9	69.0	23.2
B1	75.9	57.9	71.7	23.7
B2	76.2	59.8	73.3	23.4
B3	79.0	60.7	70.0	21.7
B4	76.7	62.4	66.6	25.4
B5	75.3	60.8	71.5	22.7
B6	78.6	66.5	75.8	20.7
B7	74.9	59.1	70.5	23.7
B8	77.0	59.6	72.5	23.4
B9	75.3	58.1	72.0	24.3
B10	75.5	57.5	73.2	25.5
B11	74.8	59.4	72.7	24.9
B12	73.6	55.2	68.6	24.9

**Fig. 4:**
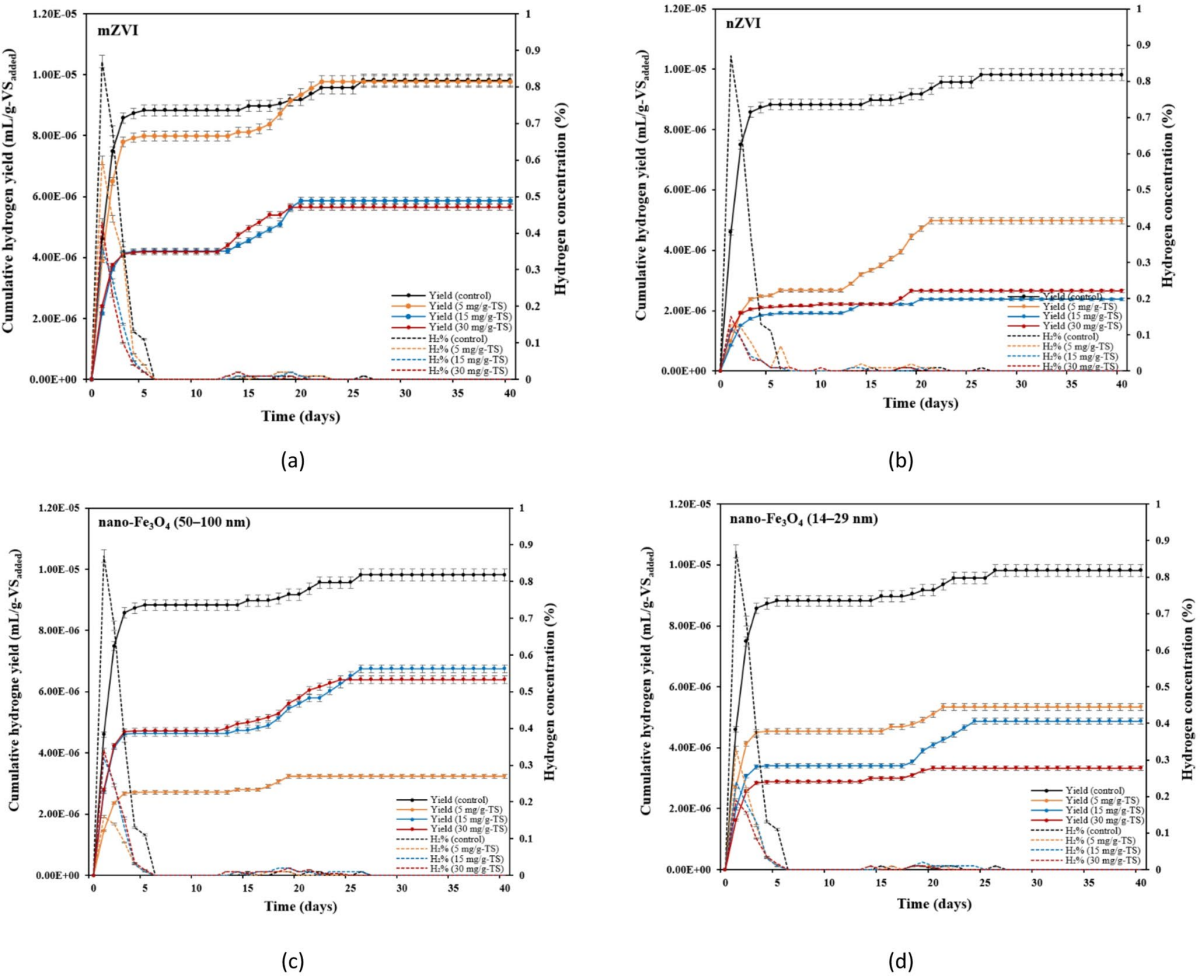
Effect of iron-based materials on cumulative hydrogen yield and hydrogen concentration under different dosages: **a** mZVI; **b** nZVI; **c** nano-Fe_3_O_4_ (50–100 nm); and **d** nano-Fe_3_O_4_ (14–29 nm)

As shown in Fig. 4, H_2_ yield decreased with the addition of all iron-based materials. The highest decrease of 76% and 73% of H_2_ yield was observed with the addition of 15 mg/g-TS and 30 mg/g-TS nZVI (Fig. 4b). It may be speculated that higher dosages of iron-based additives, particularly nZVI, promoted the growth of active hydrogenotrophic methanogens, which use H_2_ (generated by ZVI reactions) and CO_2_ to produce CH_4_. The study performed by Liu et al. [59] showed that the relative abundance of H_2_-utilizing methanogens in the total methanogen population in the reactor with ZVI treatment was almost twice as high as in the control reactor (22% and 13%, respectively). Another study confirmed that ZVI can promote hydrogen-consuming biological processes, leading to a reduction in H_2_ levels within the anaerobic system [54]. This study showed that the THSS treated with iron-based materials reached peak H_2_ production on the first day of the reaction, with values of 0.36–0.60%, 0.12–0.15%, 0.16–0.34%, and 0.19–0.33% for mZVI, nZVI, nano-Fe_3_O_4_ (50–100 nm), and nano-Fe_3_O_4_ (14–29 nm), respectively. The findings revealed that 71–80%, 48–77%, 68–83%, and 69–85% of the total H_2_ was generated within the first 3 days, indicating that H_2_ production from 5 to 30 mg/g-TS dosages of mZVI, nZVI, nano-Fe_3_O_4_ (50–100 nm), and nano-Fe_3_O_4_ (14–29 nm) occurred rapidly. An exception was observed in the group with an nZVI dosage of 5 mg/g-TS, where more than 70% of the total H_2_ volume was produced only after 16 days (Fig. 4b). Similar results have been reported by Yang et al. [57], where more than 75% of the total H_2_ is produced within the first 3 days in the presence of nZVI particles. The content of H_2_ in all examined groups gradually decreased over time, which may be related to its utilization by hydrogenotrophic methanogens to produce CH_4_ [60]. Contrary to this study, Wang et al. [52] reported that both nZVI and ZVI (100 mesh) enhanced H_2_ production during AD of WAS, with the 4 and 10 g/L nZVI dosages showing the greatest increases compared with the control. The study explained that this rapid and significant H_2_ accumulation at higher nZVI concentrations led to reduced CH_4_ production. This phenomenon occurs most often with high nZVI addition as its large SSA makes it highly reactive and capable of releasing H_2_ within a short time, which can cause a substantial H_2_ shock to the AD system.

**Fig. 5:**
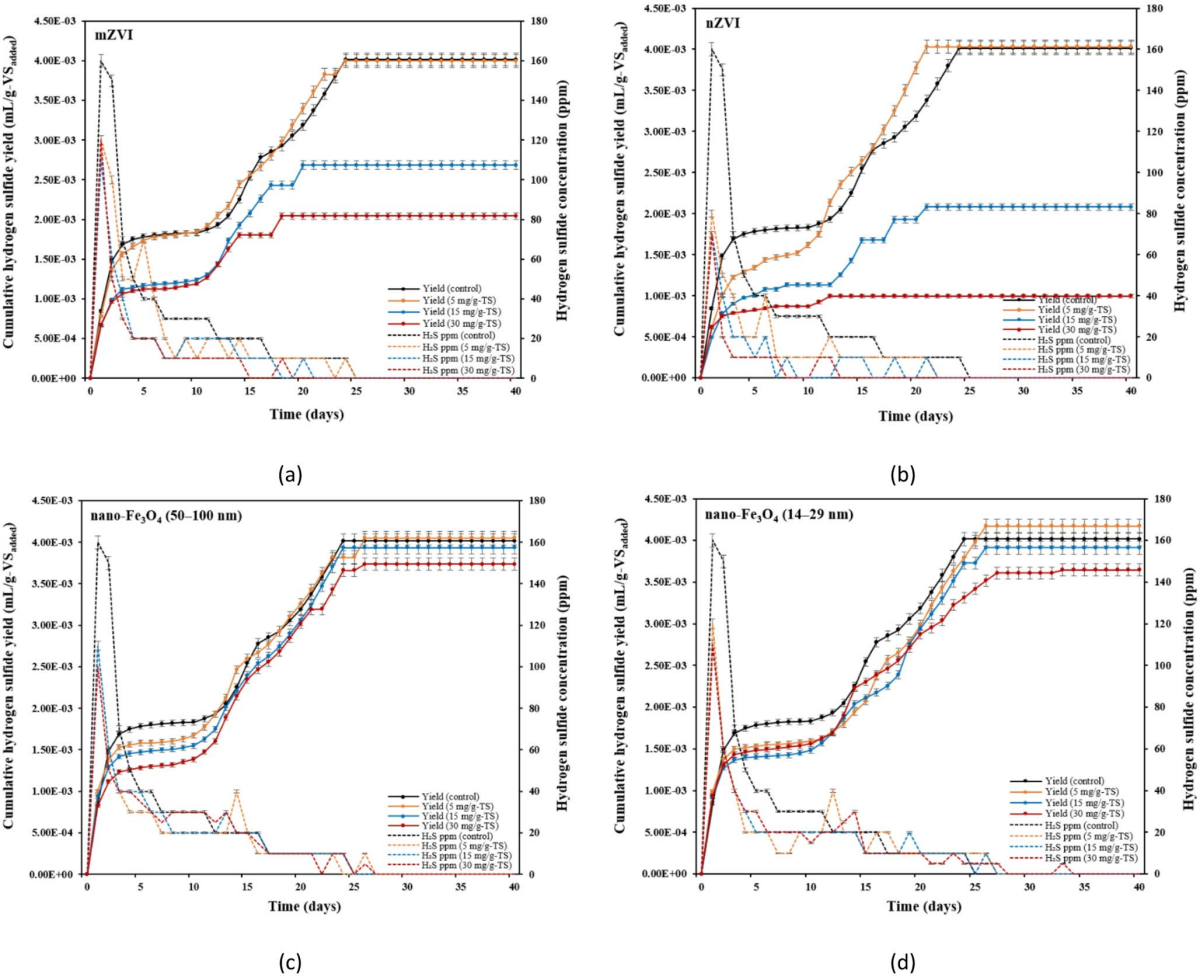
Effects of iron-based materials on cumulative hydrogen sulfide yield and hydrogen sulfide concentration at various dosages: **a** mZVI; **b** nZVI; **c** nano-Fe_3_O_4_ (50–10 nm); and **d** nano-Fe_3_O_4_ (14–29 nm)

Strictly regulating H_2_S production is crucial as it can inhibit the activity of acetogenic bacteria, suppress methanogens, and cause equipment corrosion [61, 62]. Among the different iron-based additives, nZVI was the most effective in removing H_2_S throughout the entire AD process. After 40 days of digestion, the H_2_S yield significantly decreased by 48% and 75% at nZVI dosages of 15 and 30 mg/g-TS, respectively, compared to the control (Fig. 5b). In contrast, mZVI resulted in a lower decrease in H_2_S yield at the same dosages, accounting for 33% and 49%, respectively (Fig. 5a). The smallest reduction in H_2_S compared to the control was observed with both magnetite particle sizes, reaching 2.01–6.96% and 2.59–9.18% at 15–30 mg/g-TS for the 50–100 nm and 14–29 nm particles, respectively (Fig. 5c, d). On the other hand, a slight increase in H_2_S yield was observed with almost all iron-based additives at the 5 mg/g-TS dosage (except mZVI), with the highest increase of 3.91% occurring during anaerobic sludge digestion supplemented with nano-Fe_3_O_4_ (14–29 nm). Consistent with these results, another study performed by Su et al. [63] reported that nZVI addition significantly suppressed H_2_S production. Hydrogen sulfide produced by sulfate-reducing bacteria can be immobilized with the help of nZVI through the particular reactions: Fe(0) + H_2_S = FeS + H_2_; 2FeOOH + 3H_2_S = 2FeS + 1/8S_8_ + 4H_2_O; 2FeOOH + 3H_2_S = FeS_2_ + FeS + 4H_2_O. These reactions lead to the formation of FeS, S_8_, and FeS_2_, resulting in a significant reduction of H_2_S concentration in biogas. A hydrous Fe(II)/Fe(III) oxide layer at the surface of ZVI promotes H_2_S removal and consequently reduces H_2_S concentration in biogas during AD.

### The effects of iron-based additives on TS, VS, and COD removal

**Fig. 6:**
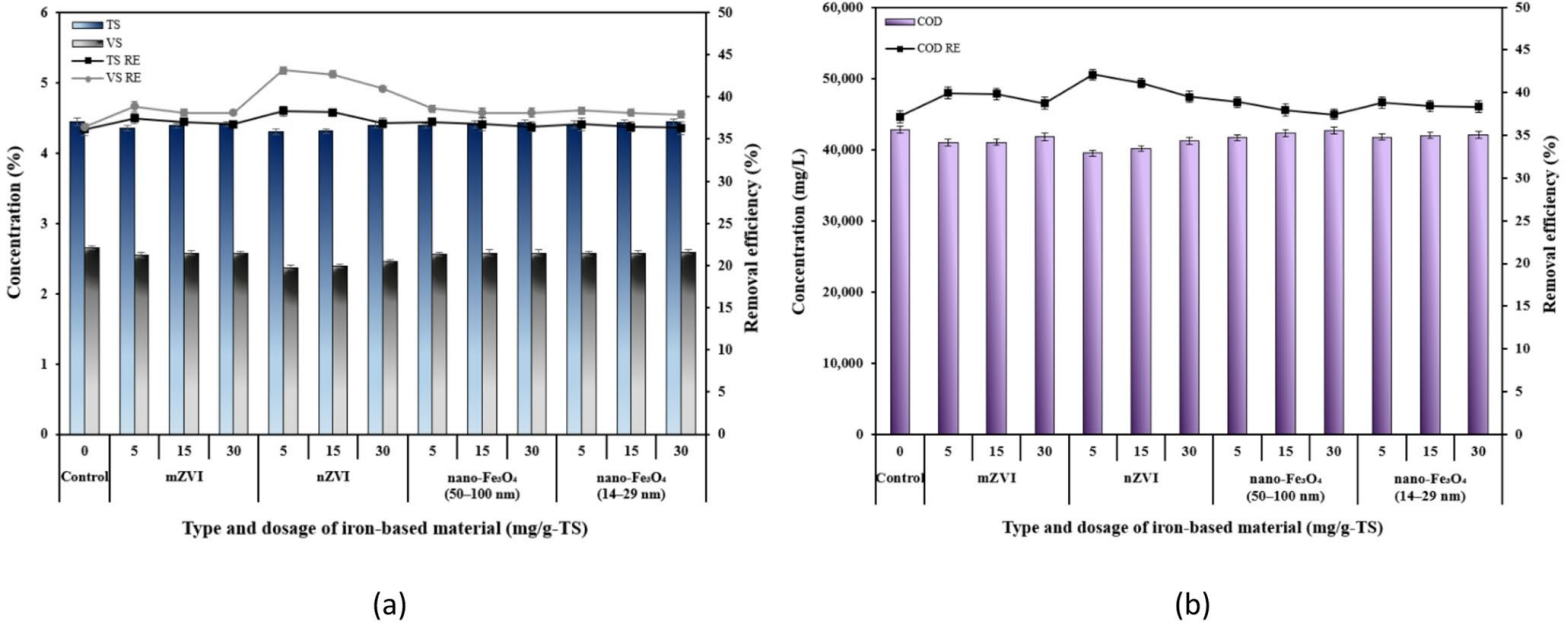
Final concentrations and removal efficiencies of **a** TS and VS and **b** COD after 40 d

The reduction in the organic fraction of THSS by different dosages of iron-based additives was determined by analyzing parameters such as COD, VS, and TS, measured before and after the AD process. The control group showed the lowest degradation rates, with 36.2% for TS and 36.5% for VS. The greatest reduction in TS and VS was achieved when the substrate was treated with 5 mg/g-TS of nZVI, resulting in removal efficiencies of 38.3% and 43.2%, respectively, and corresponded to 1.06 and 1.18 times more TS and VS removal efficiencies compared to the control (Fig. 6a). A similar high removal of TS and VS was also achieved at a nZVI dosage of 15 mg/g-TS, reaching 38.2% and 42.7%, respectively. As the nZVI dosage increased from 5 to 30 mg/g-TS, TS and VS removal efficiencies gradually decreased from 38.3% to 36.9% and from 43.2% to 41.0%, respectively. A similar pattern was also observed with other iron-based materials. Comparable VS removal results were reported in the study by Feng et al. [54], where a 20 g/L ZVI dosage removed 39.9% of VS. The improved sludge degradation not only lowered the overall sludge volume but also reduced the remaining organic content in both the solid and liquid phases.

Similar to the patterns observed in TS and VS removal, the COD removal efficiencies were highest at the dosage of 5 mg/g-TS of iron-based materials. After 40 days of AD, the COD of control group was 42.8 g/L, while for nZVI dosages of 5, 15, and 30 mg/g-TS, COD reached 39.5, 40.2, and 41.3 g/L, respectively. The final COD values in the nZVI-treated group showed a significant difference (*p* ≤ 0.05) compared to the control. It can be seen that the highest COD removal (42%) was achieved with the 5 mg/g-TS nZVI dosage. Similar removal efficiency of COD (46%) was achieved in another study, where 5 mg/g-VS nZVI dosage was used [47]. For both nano-Fe_3_O_4_ particle sizes, COD removal efficiencies were similar, reaching approximately 38% at a dosage of 5 mg/g-TS. Heikal et al. [64] similarly reported a COD removal efficiency of 40.5% at a magnetite dosage of 200 mg/L. Overall, it can be stated that the TS, VS, and COD trends were consistent with the biogas production results. Another study performed by Jia et al. [48] likewise observed that the final total biogas volume and the COD removal rate increased or decreased in parallel. The close link between biogas production and COD removal was explained by the fact that acidogenic and methanogenic bacteria utilize organic matter, such as COD, converting it first into organic acids and then into gases.

### Kinetic analysis

To assess impact of iron addition on the kinetics of CH_4_ production, the cumulative CH_4_ production data were fitted using MGM. Table 5 presents the kinetic parameters derived from the fitted CH_4_ production curves, showing excellent agreement with experimental data (*R²* > 0.94 for all tested groups). Additionally, each treatment group had NRMSE value below 5%, indicating a strong agreement and high accuracy between the observed and predicted CH_4_ yield values. The highest *P*_*max*_ values (233 mL/g-VS_added_) were obtained with the addition of 5 and 15 mg/g-TS of nano-Fe_3_O_4_ (14–29 nm). The highest *R*_*max*_ (18.7 mL/g-VS_added_/d) was observed with the addition of 15 mg/g-TS nZVI, representing a 39.5% increase compared to the control. In the case of mZVI, a positive linear relationship was observed between dosage and the *R*_*max*_, with the highest rate value (17.6 mL/g-VS_added_/d) achieved at dosage of 30 mg/g-TS, corresponding to a 31.3% increase compared to the control. Meanwhile, the lowest *R*_*max*_ (12.5 mL/g-VS_added_/d) was obtained with 30 mg/g-TS dosage of nano-Fe_3_O_4_ (14–29 nm). Comparing two nano-Fe_3_O_4_ sizes at the same dosage, higher *P*_*max*_ was achieved using smaller sized (14–29 nm) nano-Fe_3_O_4_, highlighting the advantageous effect of this size. A similar study reported that 120 mg/L of smaller sized nano-Fe_3_O_4_ (12–18 nm) achieved higher *P*_*max*_ (271 mL/g-VS_added_) than the larger particles (50–100 nm), which reached 195 mL/g-VS_added_ [8].

The lag phase (*λ*) represents the period during which microorganisms adjust to the available substrates. The shortest *λ*, which was equal to 9.29 d, was observed with 15 mg/g-TS nZVI addition (reactor B5). Zero-valent iron nanoparticles showed the greatest reduction in lag time—up to 38.1% compared to the control (Table 5). A similar reduction in lag phase from 15.7 to 9.87 days was observed in another study, where 2 g/L of waste iron powder was added to the dairy manure [65]. Meanwhile, in the case of mZVI, a negative linear relationship was observed, indicating that the lag time decreased as the mZVI dosage increased. In the case of mZVI, the greatest reduction in the lag phase, up to 24.0%, was observed at the highest particle dosage of 30 mg/g-TS. Thus, the addition of nZVI and mZVI reduced the lag phase, but with greater effectiveness in the case of nZVI. Meanwhile, both sizes nano-Fe_3_O_4_ had little influence on the lag phase, which remained similar to the control group (15 d). The lag phase of nano-Fe_3_O_4_ (50–100 nm) and nano-Fe_3_O_4_ (14–29 nm) reached 14.1–14.6 d and 14.6–14.9 d, respectively. Another study has also reported that the addition of different sizes of nano-Fe_3_O_4_ had only a minimal effect on the lag phase duration [8].

**Table 5:** Fitting parameters of methane production based on the modified Gompertz model

Reactor	*P*_max_ (mL/g-VS_added_)	*R*_max_ (mL/g-VS_added_/d)	λ (days)	*R* ^2^	RMSE	NRMSE
B0	214	13.4	15.0	0.99	5.07	2.48
B1	231	14.3	13.5	0.99	2.77	1.27
B2	224	15.8	12.5	0.99	3.66	1.67
B3	225	17.6	11.4	0.99	3.32	1.49
B4	229	16.5	10.4	0.95	6.13	2.75
B5	226	18.7	9.29	0.94	5.94	2.66
B6	228	18.0	11.6	0.95	6.65	3.00
B7	230	13.9	14.4	0.99	6.09	2.89
B8	228	14.2	14.6	0.99	5.34	2.51
B9	221	13.4	14.1	0.99	4.35	2.11
B10	233	13.6	14.9	0.99	3.05	1.42
B11	233	13.6	14.6	0.99	4.29	2.00
B12	228	12.5	14.9	0.99	4.52	2.22

Overall, the results indicate that among iron-based additives, nZVI was the most effective in enhancing AD process of THSS. nZVI improved methanogenesis by reducing the lag phase by up to 38.1% and increasing the methanogenic rate by up to 39.6%, as observed at a dosage of 15 mg/g-TS. Additionally, a dosage of 15 mg/g-TS nZVI significantly reduced the H_2_ yield, which decreased by 75.8% compared to the control. During biomethanation, nZVI has been proposed to serve a dual function: as an electron channel between bacteria and methanogens that facilitates direct interspecies electron transfer (DIET), and as an electron donor through the corrosion process [66]. More efficient DIET has been shown to provide a kinetic advantage for methanogenesis. In addition, previous studies have demonstrated that nZVI promotes hydrogenotrophic methanogenesis by generating H_2_ through the hydrogen evolution reaction [67]. Hydrogenotrophic microorganisms subsequently lower H_2_ levels by rapidly consuming it, which in turn enhances CH_4_ production. In addition, the corrosion of nZVI buffers acidity and reduces the oxidation-reduction potential, thus providing a more favorable environment for microorganisms.

### Total PTE concentrations, TCLP, and BCR test results

Table 6 shows the contents of total PTEs and P in THSS before AD and in THSS after AD with iron-based materials. It can be seen that total PTE and P concentrations increased in all anaerobically digested THSS samples compared to sludge before AD (i.e., the THSS-inoculum mixture at the start of digestion) [68]. The concentrations of Ni, Cr, Cu, and Zn in the control digested sludge increased by 56%, 44%, 11%, and 24%, respectively, compared to the sludge before AD. The apparent increase in total PTE concentrations after AD is mainly due to a concentration effect since the loss of VS during digestion reduces the amount of residual sludge while the absolute PTE content remains nearly constant, resulting in higher values on a dry-mass basis. A similar trend was observed for P, where its total concentration increased by 54% after AD from 10.7 g/kg to 16.5 g/kg. An increased concentration of PTEs in SS during AD has also been reported by other researchers, who explained that this effect results mainly from organic matter decomposition, biogas release, water loss, and the associated reduction in sludge mass [69, 70]. In the AD process, the transformation and agglomeration of PTEs are primarily driven by their precipitation into carbonates, hydroxides, and phosphates, as well as their adsorption by extracellular polymers, microbial cell walls, and solid particles [37]. Among all PTEs, Zn and Cu were the most abundant elements in the THSS samples, with concentrations ranging from 1435 to 1809 mg/kg and from 247 to 315 mg/kg, respectively. Similar trends have been observed by other researchers. For example, Aghanaghad et al. [71], Shamuyarira et al. [72] and He et al. [73] reported that concentrations of Zn and Cu in municipal SS samples collected from northwestern Iran, south Africa, and southern China, respectively, were higher than those of other studied PTEs. Similarly, Tytła [74] identified Zn and Cu as the dominant PTEs in SS throughout various processing stages. On average, Zn is the most commonly found PTE in SS [75]. Highest Zn concentration in the sludge could be explained by the large-scale usage of galvanized pipes in the cities [42].

The concentrations of PTEs in all digested sludge samples were significantly lower than the maximum allowable concentrations specified in the EU Directive for sewage sludge used in agriculture: 750−1200 mg/kg for Pb, 300−400 mg/kg for Ni, 1000−1750 mg/kg for Cu, 2500−4000 mg/kg for Zn, and Cr is not specified [76]. Hence, the digestate produced from AD of THSS with iron-based materials contained PTEs that fell within the permissible limits for its use on agricultural land in Europe.

**Table 6:** Total concentrations (mg/kg) of potentially toxic elements and phosphorus before and after anaerobic digestion of thermally hydrolyzed sewage sludge amended with iron-based materials

PTE and *P*	Before AD	After AD												
	B0	B0	B1	B2	B3	B4	B5	B6	B7	B8	B9	B10	B11	B12
Pb	35.7 ± 1.4	39.2 ± 0.8	36.7 ± 1.5	33.4 ± 0.9	37.1 ± 2.1	35.2 ± 1.6	34.5 ± 1.7	34.7 ± 1.8	35.3 ± 1.4	35.2 ± 1.1	33.4 ± 1.9	35.3 ± 1.5	34.6 ± 1.9	35.8 ± 1.7
Cr	91.2 ± 6.5	131 ± 12	133 ± 12	118 ± 11	124 ± 12	141 ± 12	152 ± 19	147 ± 14	151 ± 15	151 ± 13	140 ± 12	134 ± 13	137 ± 16	138 ± 9
Ni	62.8 ± 5.3	97.8 ± 9.4	101 ± 8	93.5 ± 5.1	98.7 ± 4.2	97.8 ± 10.9	101 ± 11	107 ± 4	109 ± 9	118 ± 9	101 ± 8	97.8 ± 4.6	94.7 ± 5.9	99.8 ± 8.5
Cu	247 ± 6	273 ± 16	275 ± 8	270 ± 17	259 ± 25	281 ± 15	307 ± 13	315 ± 22	295 ± 19	293 ± 17	271 ± 15	294 ± 17	282 ± 20	274 ± 21
Zn	1435 ± 102	1777 ± 85	1729 ± 75	1754 ± 109	1804 ± 70	1809 ± 125	1748 ± 105	1698 ± 109	1708 ± 89	1698 ± 84	1733 ± 92	1758 ± 60	1784 ± 64	1795 ± 112
P	10,654 ± 289	16,480 ± 603	16,087 ± 749	15,495 ± 538	16,802 ± 714	17,902 ± 495	17,809 ± 674	17,108 ± 711	16,942 ± 684	16,709 ± 484	16,782 ± 635	16,804 ± 557	16,904 ± 548	16,309 ± 594

**Fig. 7:**
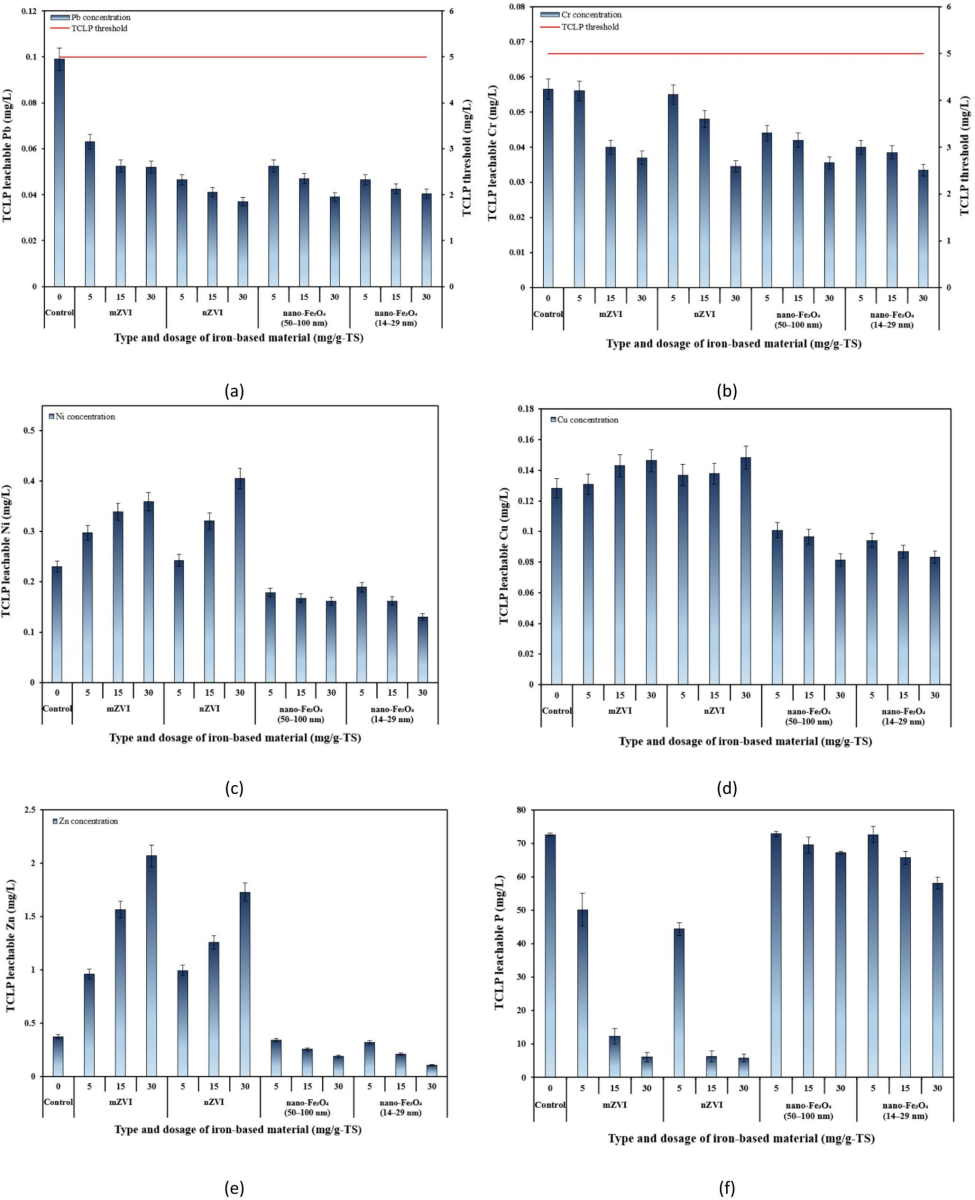
TCLP test results for digested samples treated with iron-based additives at different dosages: **a** Pb; **b** Cr; **c** Ni; **d** Cu; **e** Zn; and **f** P

The TCLP results are shown in Fig. 7. The leached concentrations of Pb and Cr were below regulatory limits (5 mg/L for both Pb and Cr), and digested sludge can be considered as ‘non-hazardous’. It can be seen that addition of 30 mg/g-TS nZVI to SS before AD greatly reduced P and Pb leachability compared to the control (by 92.1% and 62.6%, respectively) (Fig. 9f, a). The reduction in leachable P concentration through nZVI treatment is mainly driven by precipitation processes and the formation of Fe-P complexes [53]. For instance, Su et al. [63] reported via XRD analyses that nZVI facilitated the formation of vivianite (Fe_3_(PO_4_)_2_) during the AD of SS, suggesting that soluble P was immobilized through iron phosphate precipitation. Since Pb^2+^ possesses the smallest hydrated ionic radius (4.01 Å) and the greatest ability to compete with protons, it showed the highest relative adsorption capacity among studied PTEs [77]. Meanwhile, smaller sized nano-Fe_3_O_4_ (14−29 nm) at dosage of 30 mg/g-TS was the most effective in reducing the leaching of Zn and Ni (by 71.24% and 43.48%, respectively) (Fig. 7e, c). Since Zn and Ni in sludge are easily mobilized [68], they showed the highest leachability among all PTEs, and they were more susceptible to the treatment by nano-Fe_3_O_4_. Cu leaching decreased with increasing dosages of both sizes of nano-Fe_3_O_4_, with the lowest values observed in anaerobically digested THSS treated with 30 mg/g-TS nano-Fe_3_O_4_, showing reductions of 36.4% and 35.1% for 50−100 nm and 14−29 nm particles, respectively, compared to the control (Fig. 7d). On the contrary, a significant increase in the leaching of Zn and Ni, and a slight increase in the leaching of Cu from mZVI and nZVI-treated anaerobically digested sludge compared to the control was observed. The leaching of Zn increased by 5.59 and 4.68 times from the 30 mg/g-TS mZVI and nZVI groups, respectively, while Ni increased slightly less - by 1.57 and 1.78 times, respectively. Meanwhile, the leaching of Cu only slightly increased in the mZVI and nZVI groups (30 mg/g-TS) compared to the control - by 14.0% and 15.6%, respectively. In general, the leachability of PTEs from the final anaerobically digested sludge was low, indicating that the digested material is stable and poses minimal environmental risk.

According to the TCLP results, among all the studied PTEs, Pb and Cr appeared to be more effectively immobilized in the digested sludge treated with mZVI and nZVI, as indicated by their lower concentrations in the leachates compared with the control. These results are consistent with other AD and adsorption studies showing that ZVI can remove cationic PTEs (particularly Pb and Cr) from aqueous solutions through both adsorption and reduction mechanisms, since iron is a strong reducing agent. Zhang et al. [78] showed that AD of SS with the addition of 2.5 g nZVI-BC decreased the available content of Cr by the largest amount (54%), compared to the control, among the five studied PTEs. Another study by Suanon et al. [28] found that in the presence of nZVI, PTEs were better stabilized within solid digestate, as the mobile fractions were significantly reduced. It has been proposed that nZVI rapidly forms an oxyhydroxide shell in aqueous environment, which provides active sites for PTE sorption and co-precipitation. Meanwhile, nano-Fe_3_O_4_ have been reported to remove PTEs through both physical and chemical adsorption [79]. For example, Hu et al. [80] observed the formation of FeCr_2_O_4_ crystals on the surface of nano-Fe_3_O_4_, resulting from the reduction of Cr(VI) to Cr(III) followed by surface precipitation of Cr(III).

**Fig. 8:**
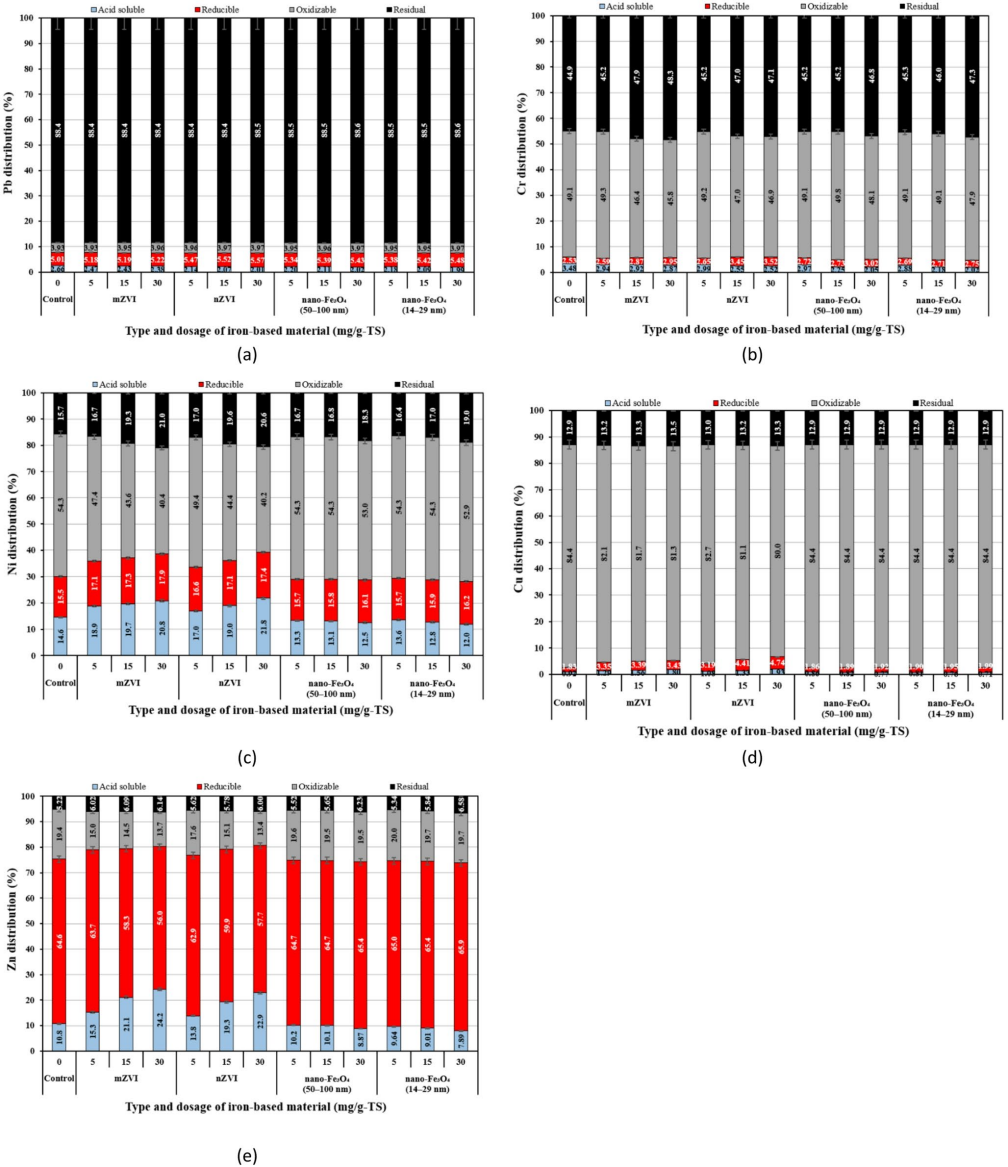
Potentially toxic element distribution in anaerobically digested thermally hydrolyzed sewage sludge amended with iron-based materials: **a** Pb; **b** Cr; **c** Ni; **d** Cu; and **e** Zn

Figure 8 presents the results of the BCR sequential extraction analysis for PTEs in the anaerobically digested THSS samples treated with iron-based additives. The distribution of PTE fractions varied for each individual PTE. Pb was mainly associated with the residual fraction (up to 88.6%, Fig. 8a), likely because it readily forms stable compounds with P and sulfur commonly present in sludge (Fig. 8a). Similar results were reported in another study, where the majority of the Pb in sludge was associated with the residual fraction [81]. The predominance of Pb in the residual fraction of the sludge digestate suggests that it is largely unavailable for plant uptake. Cr was almost evenly distributed between the oxidizable and residual fractions (up to 49.8% and up to 48.3%, Fig. 8b). Ni was primarily associated with the oxidizable fraction, followed by the residual, reducible, and acid-soluble fractions (Fig. 8c). The Cu distribution followed the following order: oxidizable > residual > reducible > acid-soluble (Fig. 8d). Cu was predominantly associated with the oxidizable fraction (80.0–84.4%) in all treatment groups. This observation agrees with previous findings [45, 82, 83], where majority of Cu was found in the oxidizable fraction of SS. The high Cu concentration in the organic fraction is likely due to its strong affinity for organic matter, which facilitates the formation of organo-metallic complexes [84]. Zn was predominantly found in the reducible fraction (up to 65.9%, Fig. 8e). Zhu et al. [38] similarly reported that Zn was mainly bound to the Fe–Mn oxide fraction in anaerobic sludge digestate. Studies on contaminated soil have also indicated that Zn is one of the most labile metals, due to its greater affinity for the non-residual fraction [85]. The relatively low concentration of Zn in the oxidizable fraction (13.4–20.0%) compared with the other elements was likely due to competition between metal–organic complexes and aqueous Fe–Mn oxide binding sites [86].

Ni and Zn were the most mobile elements in the control digested THSS sample, accounting for the highest percentage fractions among all PTEs (14.6% and 10.8%, respectively), while Cu was the least mobile (0.92%). This is consistent with the study by Dai et al. [87], which found that Ni was the most mobile element, followed by Zn. It can be seen that concentrations of Zn, Ni, and Cu in the acid-soluble fraction increased with increasing mZVI and nZVI dosage. At mZVI and nZVI dosages of 30 mg/g-TS, the Zn concentration increased by 2.25- and 2.13-fold, Ni by 1.43- and 1.50-fold, and Cu by 1.96- and 2.10-fold, respectively. Similar trends in leached PTE concentrations were also observed in the TCLP leaching test for mZVI and nZVI treated sludge samples. In contrast, it was observed that the addition of both nano-Fe_3_O_4_ particles reduced the concentrations of all studied PTEs in the acid-soluble fraction, while increasing their concentrations in the reducible and residual fractions of digested THSS samples. For example, the addition of 30 mg/g-TS of nano-Fe_3_O_4_ (50–100 nm) decreased the Zn content in acid-soluble fraction from 10.8% to 8.87%, while slightly increasing Zn in the reducible fraction from 64.6% to 65.4% and in the residual fraction from 5.22% to 6.23%. This is particularly important for the agricultural application of sludge digestate, since the reduced availability of PTEs can lower environmental risks associated with their mobility. Another study also demonstrated that nano-Fe_3_O_4_ reduced the most mobile fractions, which was attributed to physicochemical adsorption mechanisms [28].

Both the mZVI and nZVI particles notably decreased the concentrations of the Cr, Ni, Cu, and Zn in the oxidizable fraction compared to the control, with the reduction generally increasing with higher dosages of these iron-based materials. For example, the addition of mZVI and nZVI particles at a dosage of 30 mg/g-TS decreased the percentage of Ni in the oxidizable fraction from 54.3% in the control to 40.4% and 40.2%, respectively. This is likely because the ZVI treatment effectively promoted the breakdown of organic matter in the anaerobically digested sludge [88]. Other studies similarly found that nZVI decreased the percentage of PTEs in oxidizable form [28, 78].

Although ZVI has been widely applied for removing various contaminants from water and soil, little research has been conducted on how this material influences the mobility and speciation of PTEs in SS. In this study, mZVI and nZVI tended to enhance AD of THSS; however, their application increased the concentrations of Zn, Ni, and Cu in the most available (acid-soluble) fraction with increasing dosages. This effect can be attributed to the increased degradation of organic matter caused by mZVI and nZVI, which released PTEs previously associated with the oxidizable fraction. The released metals were subsequently redistributed, with Ni and Cu increasing in acid-soluble, reducible, and residual fractions, while Zn content increased only in the acid-soluble and residual fractions. For Zn, the increase in mZVI and nZVI dosages led to a decrease in Zn content in the reducible fraction, probably due to the reductive dissolution of Fe(III) oxides under lowered ORP conditions induced by the ZVI particles. This process may have released Zn that was previously bound to Fe-Mn oxides. A previous study reported that the addition of 0.5% nZVI in conventional (without thermal pretreatment) sludge enhanced CH_4_ production and simultaneously promoted the release of PTEs from the organic-bound fraction, while the concentrations of PTEs in most mobile forms decreased [28]. It was suggested that metals released from organics could be immobilized through adsorption or co-precipitation with iron oxide surfaces. In the present study, thermal hydrolysis likely enhanced the accessibility of organic matter to mZVI and nZVI by reducing sludge particle size and increasing surface area [89], thereby allowing these particles to promote organic matter degradation more effectively. This enhanced interaction between iron-based materials and organic matter resulted in a greater release of PTEs from the organic fraction. Without THP, the release of PTEs from the organic fraction would likely be less pronounced. For Pb and Cr, the addition of mZVI and nZVI led to a decrease in their content in the acid-soluble fraction. The contrasting effects of ZVI particles on PTE mobility in the sludge can be explained by the different standard potentials of Ni(II), Zn(II), and Pb(II). Ni(II) (*E*^0^ = − 0.25 V) and Zn(II) (*E*^0^ = − 0.76 V) have standard potentials that are either slightly more positive or more negative than that of Fe (*E*^0^ = − 0.44 V), making their direct reduction by ZVI inefficient. Li et al. [90] reported that Ni(II) can be immobilized on the nZVI surface through sorption and by reduction to the less toxic metallic Ni^0^. However, this reduction pathway is often hindered by the iron oxide shell surrounding nZVI. For Zn(II), earlier research indicates that immobilization commonly occurs through surface sorption or the formation of surface complexes [91]. The previous studies suggest that the interaction of Ni and Zn with nZVI occurs predominantly through sorption onto iron oxides. However, in AD conditions, such processes appear insufficient to effectively immobilize these PTEs, which may explain their increased mobility observed in this study. In contrast, Pb(II) (*E*^0^ = − 0.13 V) has a more positive standard reduction potential than Fe^0^, making its reduction by ZVI more thermodynamically favorable and explaining better immobilization observed in this study. The immobilization of Pb in the sludge digestate can be attributed not only to reduction, but also to adsorption and precipitation. Zhang et al. [92] demonstrated that hydroxyl groups arising from Fe(OH)_3_ on the nZVI shell provided abundant binding sites for Pb(II) adsorption. Several studies have similarly shown that the immobilization of Zn(II) by nZVI in different media, including soil, is significantly lower than that of Pb(II) [91, 93]. This has been explained by the combined effects of reduction and sorption in the case of Pb(II), whereas Zn(II) and Ni(II) can be immobilized only through surface sorption [90, 93].

**Fig. 9:**
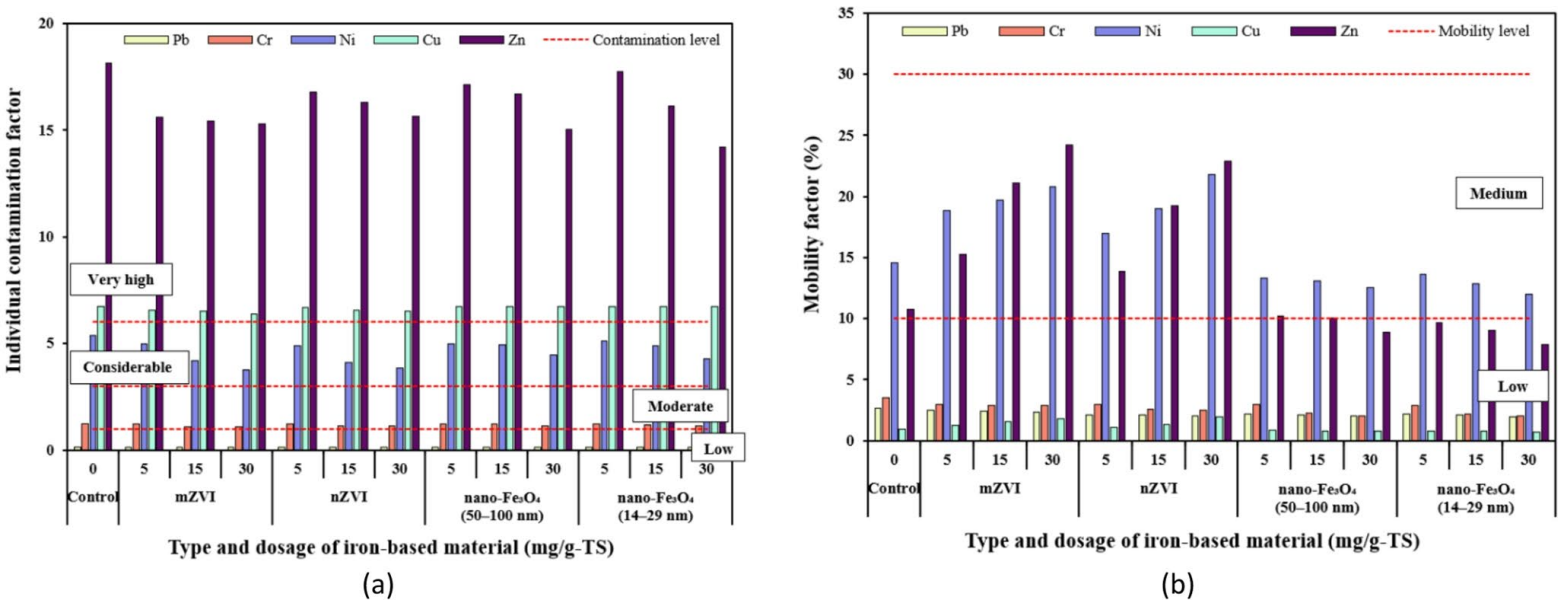
Results of potentially toxic elements in anaerobically digested thermally hydrolyzed sewage sludge amended with iron-based additives based on: **a** individual contamination factor (ICF) and **b** mobility factor (MF)

According to the average ICF values, anaerobically digested THSS, whether amended with iron-based materials or not, showed very high contamination with Zn and Cu and considerable contamination with Ni (Fig. 9a). Increasing the particle dosage led to a decrease in the contamination factors of Zn and Ni, indicating a linear inverse relationship. A 30 mg/g-TS dosage of nano-Fe_3_O_4_ (14–29 nm) appeared to be most effective in reducing Zn contamination in the digestate compared to the other iron-based material types, as indicated by considerably lower ICF value (14.2) relative to the control (18.1).

Given the low percentage of studied PTEs in the F1 fraction (representing the most mobile form), Pb, Cr, and Cu were not mobile in any of the treatment groups and can be considered non-mobile (Fig. 9b). Meanwhile, Ni was moderately mobile in all of the treatment groups, while Zn was moderately mobile in almost all of them—that is, except in the THSS treated with nano-Fe_3_O_4_ (50–100 nm) at a dosage of 30 mg/g-TS and nano-Fe_3_O_4_ (14–29 nm) at dosages of 5–30 mg/g-TS. Similar to the contamination assessment, the mobility analysis indicated that nano-Fe_3_O_4_ was effective in reducing both the contamination levels and mobility of Zn and Ni in anaerobically digested THSS. Magnetite nanoparticles sized 14–29 nm at a dosage of 30 mg/g-TS led to the greatest reduction in Zn mobility from moderately mobile (10.8% in the control) to non-mobile (7.89%). A similar effect was observed for Ni, for which the MF value decreased from 14.6% in the control to 11.9% in reactor B12 when the same size and dosage of magnetite nanoparticles were used. It is known that iron oxides can bind metals, thereby decreasing their bioavailable fraction [94]. On the other hand, it can be seen that mZVI and nZVI had a negative effect on the immobilization of Zn and Ni. A linear positive relationship was observed between the increase in Zn and Ni mobility factors and the increasing dosages of mZVI and nZVI. Zn and Ni exhibited the greatest MF values (> 20%) in reactors B3 and B6, suggesting that these PTEs have strong potential for leaching from the anaerobically digested THSS treated with 30 mg/g-TS of mZVI and nZVI. It also suggests a potential environmental risk, particularly to ecosystems, if such sludge is repeatedly applied to agricultural soils [28]. The results showed that Zn poses the greatest environmental risk among all the PTE examined, owing to its high concentration in the bioavailable (acid-soluble and reducible) fraction and its inherent elemental toxicity. Ni was identified as the second most toxic element in this study as it exhibited lower contents in the acid-soluble and reducible fractions compared to Zn, but higher than those of other elements. Due to the toxic effects of Zn and Ni on plant growth, the application of such sludge digestate in agriculture should be carefully considered [95].

This study investigated the leaching behaviour and metal speciation in THSS digestate to predict the potential fate of PTEs during final disposal, either in landfills or agricultural applications, and to evaluate how the addition of iron-based materials influences their mobility. The primary constraint on the agricultural application or landfilling of sludge is the presence of PTEs, whose overall contamination is often assessed by comparing their total concentrations with the regulatory limit values established in national or international guidelines. However, total concentrations of PTEs often fail to represent the actual environmental risk as they do not consider the specific metal fractions that may potentially be released into soils and groundwater. For this reason, in order to effectively assess the potential soil contamination by PTEs resulting from digestate application, pollution indices such as ICF and MF were used. ICF reflects both the environmental risk and persistence of PTEs, indicating their potential release into water bodies [96]. Meanwhile, MF represents the proportion of the most bioavailable and mobile metal fraction in the sediments and organic amendments, which can pose a potential risk to the environment [44]. Therefore, both ICF and MF can be collectively referred to as environmental risk indices, which assess ecological risk, PTE mobility in environmental matrices, and their bioavailability to living organisms. In this study, Ni, Cu, and Zn consistently exhibited the highest ICF values across all sludge digestate treatments, with Ni (3.77–5.38) falling into the ‘considerable’ category, while Cu (6.39–6.75) and Zn (14.2–18.1) were classified in the ‘very high’ contamination category. In soils, elevated ICF values indicate a greater likelihood of PTE leaching to groundwater or uptake by crops, which may result in food chain contamination and long-term soil quality deterioration. Another study performed by Tytła [74] similarly found that according to ICF, SS was highly polluted with Zn (14.4–63). In contrast, this study showed that there was very low pollution of the THSS digestate with Pb due to low the ICF values (≈ 0.13). This shows that this PTE was mainly concentrated in immobile form and does not produce toxicological effects under different environments. This is further supported by MF values, which showed that Pb, along with Cr and Cu, remained below the threshold value (≤ 10%) for low-risk mobility. When compared to the threshold MF value, Zn and Ni consistently exceeded this limit across all ZVI treatments, highlighting their high mobility and potential environmental risk. In contrast to ZVI, nano-Fe_3_O_4_ markedly reduced the MF values for Zn and Ni, with higher dosages bringing them close to or below the threshold value. This suggests that nano-Fe_3_O_4_ promotes the immobilization of these PTEs, likely due to its stable crystalline structure [97].

Since the MF values for Pb, Cr, and Cu were below 10% in all treatment groups, this indicates that these PTEs exhibited high stability in the digested sludge regardless of the iron additive applied. It can be observed that in the case of Ni, all treatment groups showed a medium mobility risk (11% ≤ MF ≤ 30%); whereas for Zn, the 30 mg/g-TS dosage of nano-Fe_3_O_4_ (50–100 nm), as well as all dosages of nano-Fe_3_O_4_ (14–29 nm), led to stabilization of this PTE, reaching a low mobility risk (MF ≤ 10%). There is also a clear trend that higher nZVI dosages increased the mobility risk of both Ni and Zn; while in the case of small-sized nano-Fe_3_O_4_ particles, the opposite trend was observed. This demonstrates that magnetite addition during AD of THSS can reduce the mobility risk of these PTEs, and that smaller particle sizes, regardless of the tested dosage, may lower Zn mobility risk to a minimal level. However, regarding methane yields, the opposite trend was observed between the different iron particles: nZVI particles provided the highest improvement in methane yield (up to 9.31% at 5 and 15 mg/g-TS dosages), whereas the smaller nano-Fe_3_O_4_ particles (14–29 nm, at 30 mg/g-TS) had no effect on cumulative methane yield compared to the control. A similar trend to the CH_4_ yield results was observed in the energy gain from CH_4_, where nZVI dosages of 5 and 15 mg/g-TS led to the highest *E*_*CH4*_ value (479 kJ), while the 30 mg/g-TS dosage of nano-Fe_3_O_4_ (14–29 nm) resulted in the lowest *E*_*CH4*_, which was the same as that of the control (438 kJ). Overall, the results indicate that the addition of iron-based particles during AD influences both PTE stability and CH_4_ production. While nano-Fe_3_O_4_, particularly in smaller particle sizes (14–29 nm), effectively reduced the mobility risk of Zn (MF ≤ 10%), nZVI had the strongest positive effect on methane yield and energy recovery. Thus, there is a trade-off: improving PTE stabilization tends to be more pronounced with nano-Fe_3_O_4_, whereas maximizing CH_4_ generation and energy gain is more strongly linked to nZVI addition.

These results indicate that a two-stage dosing strategy—using nZVI at start-up to accelerate CH_4_ production followed by nano-Fe_3_O_4_ addition during the stabilization phase to reduce Zn and Ni mobility—may help balance energy recovery with environmental safety. Future works should systematically optimize this approach, including the timing of the transition between stages, the specific dosages of nZVI and nano-Fe_3_O_4_, and the criteria for determining when to shift from nZVI to nano-Fe_3_O_4_ application (e.g., at peak CH_4_ production rate, based on VFA profiles, or according to risk indices). Long-term continuous trials and scale-up studies will also be required to validate the sustainability of this strategy prior to practical application.

## Conclusions

This study investigated the effects of different iron-based materials, including mZVI, nZVI, and two differently sized nano-Fe_3_O_4_ particles, on the AD performance and PTE speciation in THSS. Among the tested materials, nZVI was the most effective at enhancing CH_4_ production. The optimal dosage of 15 mg/g-TS increased cumulative CH_4_ yield by 9%, reduced the lag phase by 1.6-fold, and enhanced the maximum CH_4_ production rate by 1.4-fold. In contrast, both sizes of nano-Fe_3_O_4_ particles had little or no effect on CH_4_ production. However, nZVI particles increased the mobility of Zn, Ni, and Cu in digested THSS, while higher dosages of smaller-sized nano-Fe_3_O_4_ were most effective in immobilizing PTEs, particularly Zn, reducing its mobility factor from a medium to a low level. These results demonstrate that the choice of iron-based material dictates the trade-off between enhanced biogas production and reduced environmental risk associated with PTEs in digested SS. Therefore, nZVI is recommended as an additive for maximizing energy recovery, whereas nano-Fe_3_O_4_ shows great promise as a stabilizing agent for PTEs in sludge intended for agricultural use, mitigating the risk of PTE leaching. A limitation of this study is that only THSS was investigated, which may contain inhibitory compounds that can negatively affect microorganisms during the initial phase of AD. Since this study demonstrated the beneficial effect of nZVI on THSS AD, future studies should conduct comparative investigations of the effects of different iron-based materials on CH_4_ production and PTE transformations in both untreated and THSS, integrating complementary analyses, such as VFA dynamics and microbial community profiling, to provide a more comprehensive understanding on how these materials influences AD.

## Data Availability

Data will be made available on request.
